# Overlapping Post-COVID Conditions: A Case of Myalgic Encephalomyelitis and Postural Orthostatic Tachycardia Syndrome Triggered by Sequential Epstein-Barr Virus (EBV) and Severe Acute Respiratory Syndrome Coronavirus 2 (SARS-CoV-2) Infections

**DOI:** 10.7759/cureus.116844

**Published:** 2026-09-24

**Authors:** Virgínia R Mayrink Magalhães, Laura A Ribeiro Oliveira, Adelina J Lourenço, Isabela Ribeiro Camargos, Lucas G Da Silva, Priscilla S Filgueiras, Ana E De Souza Lima, Sarah V Marques Contin, Marcela Costa, Plinio Istilli, Marcelo Ferreira da Costa Gomes, Camila B Pedretti, Paulo Roberto Lopes Correa, Rafaella F Grenfell

**Affiliations:** 1 Diagnosis and Therapy of Infectious Diseases and Cancer, Oswaldo Cruz Foundation, Rio de Janeiro, BRA; 2 Molecular Medicine, Federal University of Minas Gerais, Belo Horizonte, BRA; 3 Pediatric Infectious Diseases, Ministry of Health, Brasília, BRA; 4 Vigilance of COVID-19, Influenza and Other Respiratory Viruses, Ministry of Health, Brasília, BRA; 5 Scientific Computing, Oswaldo Cruz Foundation, Rio de Janeiro, BRA; 6 Vigilance in Health and Environment, Department of Transmissible Diseases, Ministry of Health, Brazilia, BRA; 7 Nursing, Belo Horizonte Municipal Health Department, Belo Horizonte, BRA; 8 Health, Belo Horizonte Municipal Government, Belo Horizonte, BRA

**Keywords:** myalgic encephalomyelitis/chronic fatigue syndrome (me/cfs), post-covid conditions, postural orthostatic tachycardia syndrome (pots), sars-cov-2, sequential viral infection

## Abstract

The rise in post-infectious syndromes following COVID-19, such as Myalgic Encephalomyelitis/Chronic Fatigue Syndrome (ME/CFS) and Postural Orthostatic Tachycardia Syndrome (POTS), has become increasingly common in clinical practice, representing an emerging clinical challenge. This case highlights a possible clinical scenario involving the interaction between sequential viral infections - such as the Epstein-Barr virus (EBV) and Severe Acute Respiratory Syndrome Coronavirus 2 (SARS-CoV-2) - in triggering overlapping chronic conditions. This report presents the case of a patient who developed ME/CFS and POTS following SARS-CoV-2 infection, with a prior history of EBV mononucleosis, and discusses the diagnostic, therapeutic, and pathophysiological implications. A 48-year-old woman with a history of mononucleosis in childhood developed persistent and disabling symptoms after COVID-19 in January 2022. She presented with extreme fatigue, orthostatic intolerance, paresthesia, cognitive dysfunction, and multisystem symptoms. After extensive investigation, she was diagnosed with ME/CFS (early 2023) and POTS (later that year). Mast Cell Activation Syndrome (MCAS) was considered as a hypothesis during follow-up, but objective testing was not confirmatory. Management included pharmacological therapy (including low-dose naltrexone, duloxetine, and palmitoylethanolamide (PEA)), activity adjustments (pacing), and a multidisciplinary approach, with partial and fluctuating responses. This case illustrates how viral infections such as EBV and SARS-CoV-2 may act as sequential triggers in an individual clinical trajectory compatible with the development of ME/CFS and POTS. The symptomatic overlap, the lack of readily available objective biomarkers in this patient, and the need for a predominantly clinical and functional diagnostic approach in her evaluation highlight the complexity of these conditions. This experience also underscores the importance of continued efforts in research, clinical training, and integrated care models to improve the management of patients presenting with post-COVID conditions (PCC).

## Introduction

Myalgic Encephalomyelitis, also referred to as Chronic Fatigue Syndrome (ME/CFS), is a debilitating condition characterized by persistent and disabling fatigue without apparent cause, which does not improve with rest and worsens after physical or cognitive exertion. Post-exertional malaise (PEM) is a core symptom, often accompanied by cognitive impairment, musculoskeletal pain, and sleep disturbances, resulting in a significant impact on quality of life. The disease is more prevalent in women of reproductive age, with its main risk factors including previous viral infections (such as Epstein-Barr Virus (EBV) and Severe Acute Respiratory Syndrome Coronavirus 2 (SARS-CoV-2), positive family history, autoimmune diseases, intense physical or psychological stress, and associated chronic conditions such as fibromyalgia, Ehlers-Danlos syndrome, and Postural Orthostatic Tachycardia Syndrome (POTS) [1,2].

On the other hand, POTS is a form of dysautonomia that frequently coexists with ME/CFS. It is characterized by orthostatic intolerance associated with an excessive increase in heart rate upon assuming an upright position, resulting in symptoms such as dizziness, palpitations, fatigue, and cognitive deficits. The overlap between ME/CFS and POTS is common and is associated with greater symptom intensity and worse clinical prognosis. Among the risk factors for POTS are a family history of dysautonomia, genetic predisposition, hormonal changes, and previous infections, such as mononucleosis (EBV), influenza, Lyme disease, and, more recently, COVID-19 [3-5].

These conditions share risk factors such as previous viral infections, genetic predisposition, and autoimmune diseases, suggesting an interaction between genetic susceptibility and environmental triggers [3-6].

Post-COVID conditions (PCC) are complex and multifaceted issues that arise after the acute phase of a SARS-CoV-2 infection. They are characterized by a diverse spectrum of new, recurrent, or persistent symptoms affecting multiple organ systems -such as neurological, cardiovascular, respiratory, and muscular - and which cannot be attributed to other diagnoses [7,8]. In addition to physical symptoms, studies report that many patients face intense post-pandemic emotional stress, exacerbated by uncertainty, isolation, and the burden of chronic illness [9,10]. There is also evidence that persistent inflammation may last for years after the initial infection, possibly contributing to prolonged symptomatology [11,12]. These symptoms typically manifest or persist for at least three months after the initial infection [7,8,13]. Beyond the physical impact, the condition carries profound mental, psychological, social, and economic consequences, including reduced work capacity, isolation, and diminished quality of life. Although its detailed pathophysiology remains under investigation, PCC represent a significant public health challenge, requiring a multidisciplinary approach to diagnosis, management, and support.

Objective

To report the case of a patient diagnosed with Myalgic Encephalomyelitis and Postural Orthostatic Tachycardia Syndrome who, following SARS-CoV-2 infection, developed persistent neurological, pulmonary, cardiac, cutaneous, and autonomic manifestations, resulting in significant functional impairment and reduced quality of life.

## Case presentation

The female patient had a history of six pregnancies, two deliveries, and four miscarriages. She was 48 years old, mixed-race, profession educator, and had a prior history of mononucleosis at age 11, hepatitis A at age 15, and an asthma diagnosis at age 20. The patient reported allergies to pineapple, kiwi, grapes, rosemary, oregano, eggplant, latex, shrimp, and other seafood. She experienced an episode of thrombophlebitis in her left upper limb at age 41. She denied any comorbidities prior to SARS-CoV-2 infection, as well as alcohol use and smoking. She engaged in regular physical exercise. The patient's clinical and temporal trajectory, from the acute SARS-CoV-2 infection through the evolution of PCC, diagnostic milestones, and therapeutic interventions, is detailed in Figure 1, which provides a graphical timeline of the case progression.

**Figure 1:**
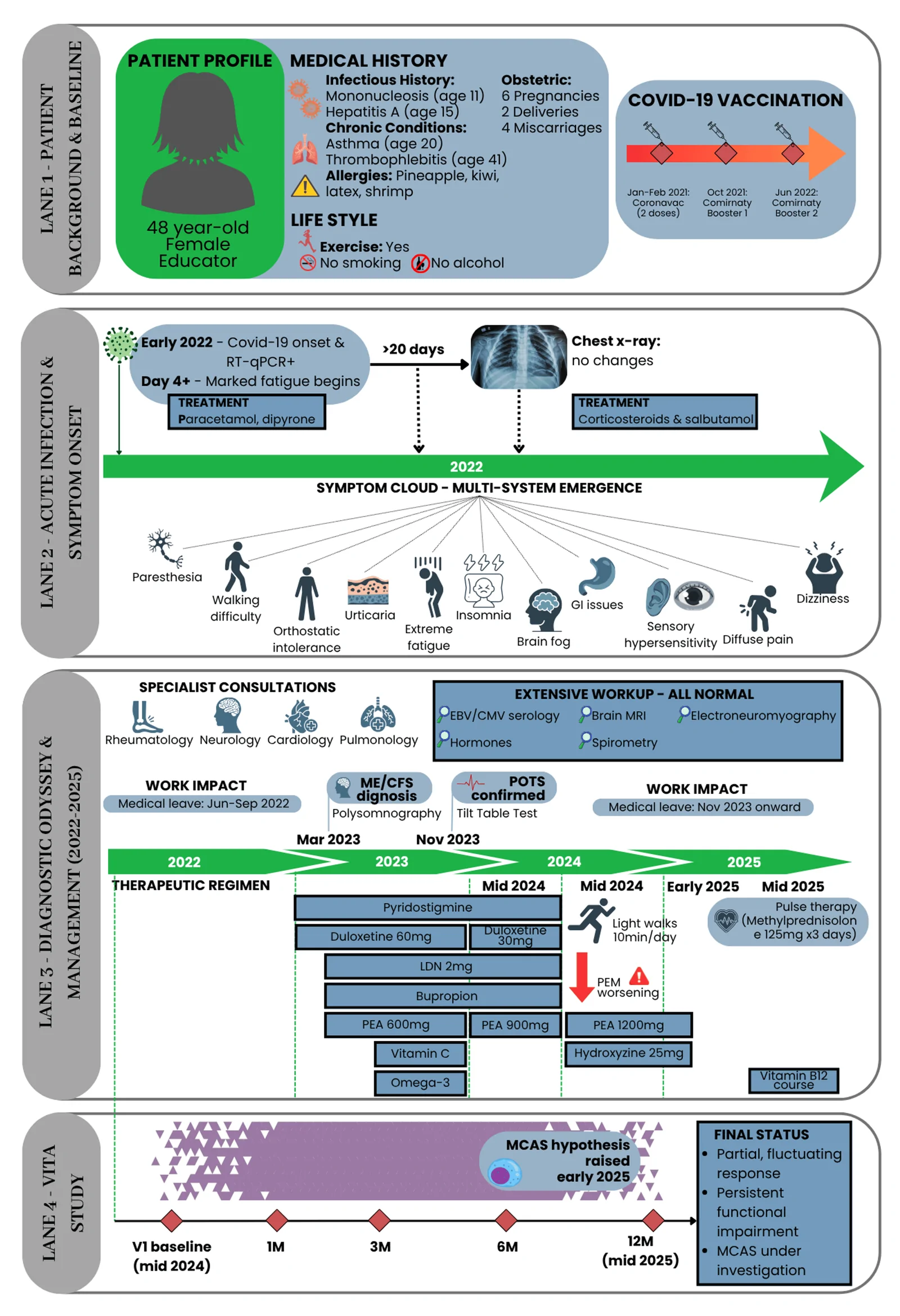
Clinical timeline and therapeutic interventions following SARS-CoV-2 infection The figure summarizes the patient's medical history, COVID-19 vaccination timeline, symptom emergence, diagnostic evaluations, and therapeutic interventions following SARS-CoV-2 infection in early 2022. Key elements include the sequential diagnoses of ME/CFS more than a year later and POTS in late 2023. Despite extensive investigations yielding normal results, multi-system symptoms persisted, leading to the hypothesis of mast cell activation syndrome (MCAS) in early 2025. The therapeutic regimen evolved over time, incorporating pyridostigmine, duloxetine, low-dose naltrexone (LDN), palmitoylethanolamide (PEA), and pulse corticosteroid therapy. Clinical monitoring was conducted as part of the VITA study cohort, with final assessment in mid-2025 indicating partial, fluctuating symptom response and persistent functional impairment. CMV, cytomegalovirus; EBV, Epstein-Barr virus; LDN, low-dose naltrexone; ME/CFS, Myalgic Encephalomyelitis/Chronic Fatigue Syndrome; MRI, magnetic resonance imaging; POTS, Postural Orthostatic Tachycardia Syndrome; RT-qPCR, reverse transcription quantitative polymerase chain reaction. Image credit: Created by the authors using Canva (Syndney, Australia).

The patient received the primary COVID-19 vaccination series with two doses of the Coronavac vaccine (SinoVac Biotech/Instituto Butantan) in early 2021. Subsequently, the first booster dose with the Comirnaty vaccine (Pfizer/BioNTech) was administered towards the end of the year followed by the second booster dose with the same vaccine in the middle of the next year. Thus, the patient completed the vaccination protocol, consisting of the primary series followed by two boosters.

During the first consultation with our team on after two years at the Carlos Prates Health Center, the patient reported that her SARS-CoV-2 infection symptoms began more than two and half years earlier. They were initially mild and characterized by nasal congestion, malaise, and dry cough. COVID-19 was confirmed by RT-qPCR. Starting on the fourth day of symptoms, she experienced marked fatigue. During the acute phase, she used paracetamol and dipyrone sporadically. Due to the persistence of symptoms beyond 20 days, additional tests were requested, including a chest X-ray, which showed no changes compared to the initial presentation. At that time, corticosteroids and salbutamol were prescribed.

New symptoms progressively emerged over the following weeks and months, including paresthesia in the lower and upper limbs, difficulty walking, sweating, cold extremities, bruising, urticaria, extreme physical and mental fatigue, insomnia, orthostatic intolerance, headache, digestive problems, shortness of breath while speaking and eating, intolerance to sound and light, low back pain, blurred vision, bladder pain, and dizziness.

The patient sought specialized care in various fields, including Rheumatology, Neurology, Infectious Diseases, Pulmonology, Internal Medicine, Cardiology, Angiology, and Chinese Medicine. Most consultations were conducted in the private healthcare network, with respiratory rehabilitation carried out at the Sarah Kubitschek network in Belo Horizonte, Brazil. Several complementary tests were performed without detecting significant abnormalities, such as laboratory tests (renal and hepatic enzyme levels, electrolytes, serology for herpes virus, cytomegalovirus, and Epstein-Barr virus, as well as urinalysis), function and imaging studies (spirometry, electroneuromyography, and brain magnetic resonance imaging), and other investigations (stool tests, pituitary hormone levels, thyroid hormones, and vitamin levels), all with results within normal parameters. Alternative diagnoses were systematically excluded through laboratory, imaging, and functional evaluations, all within normal limits.

The PCC that had persisted for over three years since the acute SARS-CoV-2 infection included orthostatic intolerance, persistent fatigue, memory impairment, paresthesia in the lower and upper limbs, sweating and cold extremities, blurred vision, sensory hypersensitivity, insomnia, low back pain, bladder pain, gastroesophageal reflux, reduced hearing, and hair loss.

The patient received diagnoses of PCC, i.e., ME/CFS in early 2023 and POTS at the end of the year. The diagnosis of ME/CFS was established according to the Institute of Medicine (IOM) 2015 diagnostic criteria (Systemic Exertion Intolerance Disease, SEID) [14]. The patient fulfilled all core criteria: (1) substantial reduction in pre-illness levels of occupational, educational, social, or personal activities, persisting for more than six months; (2) post-exertional malaise (PEM), documented by symptom worsening after light walking (10 minutes/day); (3) unrefreshing sleep; and (4) cognitive impairment and orthostatic intolerance, both present. POTS was confirmed by a head-up tilt-table test performed at an external cardiology service. Based on the medical report, the protocol involved head-up tilt at 70° for a passive phase of approximately 10 minutes. The diagnostic criterion applied was a sustained heart rate increment of ≥30 bpm within 10 minutes of upright posture, in the absence of orthostatic hypotension, accompanied by reproduction of the patient's orthostatic symptoms. Resting heart rate measurements obtained during subsequent follow-up visits were recorded as vital signs and were not used to establish the diagnosis of POTS.

Regarding work, she was on medical leave for 45 days in mid-2022, returning towards the end of the same year after symptom stabilization. A year later, due to worsening condition, she was again placed on medical leave. Her therapeutic regimen included pyridostigmine bromide 60 mg (every 12 hours), duloxetine hydrochloride 60 mg/day, naltrexone 2 mg/day, bupropion 300 mg/day, palmitoylethanolamide (PEA) 600 mg/day, vitamin C 1 g/day, and Omega-3.

During the second consultation with our team another year later, the patient reported that a rheumatologist in Belo Horizonte, consulted two months earlier, had reduced duloxetine hydrochloride to 30 mg and increased PEA to 900 mg/day, and that she experienced worsening of symptoms after starting light walks (10 minutes/day). In the third consultation two months later, the patient reported another visit to the rheumatologist (end of the year), who increased PEA to 1200 mg/day, and to a psychiatrist (end of the year), who maintained bupropion 300 mg/day, naltrexone 2 mg/day, and duloxetine hydrochloride 30 mg/day. She reported partial improvement in fatigue, orthostatic symptoms, and memory, but worsening of hair loss. She discontinued acupuncture, started hydroxyzine hydrochloride 25 mg/day for gastric and post-exertional symptoms, and continued the walks.

During the fourth consultation early next year, a worsening of her condition was observed following a trip, with increased physical and cognitive fatigue, reduced domestic and leisure activities, and more frequent flu-like symptoms. Mast Cell Activation Syndrome (MCAS) was considered as a diagnostic hypothesis, but no objective testing was available to confirm it; therefore, it remains speculative and unconfirmed. The patient consulted a new rheumatologist in São Paulo, Brazil, who requested tests and proposed pulse therapy, as well as an infectious disease specialist next month, who maintained the physiotherapy sessions.

In the last consultation with our team during monitoring in mid-2025, the patient reported starting pulse therapy with methylprednisolone 125 mg/day for three days, initiated on five months earlier, and treatment with vitamin B12 1 mg/day two months earlier.

The patient was prospectively evaluated and followed in person by the authors at our facility, and the information in this report was obtained during these consultations, complemented by review of medical records. Detailed data obtained during the clinical consultations and follow-up visits are provided in Table 1.

**Table 1:** Longitudinal anthropometric, vital signs, and handgrip strength measurements for the patient across study visits Table showing repeated measurements collected from the patient at baseline and follow-up visits (1, 3, 6, and 12 months). Variables include anthropometry (weight, height, BMI, body fat, skeletal muscle, metabolic age), vital signs (respiratory rate, heart rate, oxygen saturation, blood pressure, and temperature), dominant arm, and handgrip strength measured three times for each arm, with calculation of the mean value (kgf). Missing cells indicate measurements not performed at that visit. Changes in anthropometric and body-composition measurements are presented descriptively and should not be interpreted as attributable to physical activity, medication, or other interventions. Changes in handgrip strength are also presented descriptively; given the limited number of measurements and potential measurement variability, they should not be interpreted as evidence of a treatment effect.

Event Name	Baseline (V1)	Follow-up of 1 month (1M)	Follow-up at 3 months (3M)	Follow-up at 6 months (6M)	Follow-up at 12 months (12M)
Weight (kilograms - kg)	64.4	63.4	63.3	67.3	67.2
Height (meters - m)	1.69	1.69	1.69	1.69	1.69
Body Mass Index (BMI) (kg/m²)	22.5	22.2	22.2	23.6	23.5
Body fat (%)	34.4	34.3	32.8	37.4	29.8
Skeletal muscle (%)	26.8	26.5	27.7	25.4	30.7
Metabolic age (years)	39	38	37	43	41
Respiratory rate (breaths/min)	22	20	18	21	20
Heart rate (beats per minute - bpm)	96	79	87	79	81
Oximetry (peripheral oxygen saturation - SpO₂ %)	98	98	98	98	95
Blood pressure (millimeters of mercury - mmHg)	120x70	100x70	100x70	135x92	120X80
Temperature (degrees Celsius - °C)	35.4	34.2	34.4	36.3	35.8
Which arm is dominant? (categorical - Right / Left)	Right arm	Right arm	Right arm	Right arm	Right arm
1st right arm muscle strength (kilograms-force - Kgf)	18			16	16
2st right arm muscle strength (Kgf)	11			12	19
3st right arm muscle strength (Kgf)	10			12	16
Average right arm strength (Kgf)	13			13	17
1st left arm muscle strength (Kgf)	12			12	13
2st left arm muscle strength (Kgf)	14			16	12
3st left arm muscle strength (Kgf)	12			11	14
Average left arm strength (Kgf)	13			13	13

These consultations were conducted as part of a clinical study called VITA Monitoring, which is responsible for following 212 individuals with PCC under the coordination of the Oswaldo Cruz Foundation and in partnership with the Brazilian Ministry of Health and the Belo Horizonte Municipal Health Department. The consultations took place at the Carlos Prates Health Center in Belo Horizonte, Minas Gerais, Brazil. VITA Monitoring is an 18-month cohort with five in-person consultations and telephone follow-ups, including clinical and laboratory analyses. This work reports follow-up data at multiple time points: baseline (V1) and evaluations after one month (1M), three months (3M), six months (6M), and twelve months (12M), conducted from mid-2024 onwards for a year.

Written informed consent for publication of this case report and any accompanying images was obtained from the patient.

## Discussion

This case illustrates the complex interaction between viral infections, individual predisposition, and the emergence of chronic overlapping syndromes such as ME/CFS and POTS. The patient had a significant history of prior EBV infection in childhood, a well-established risk factor for the development of post-infectious diseases. Subsequently, the acute SARS-CoV-2 infection acted as a potent secondary triggering event, generating a cascade of persistent and debilitating symptoms. This scenario supports the double-hit vulnerability model, in which an initial viral infection predisposes the immune and neurological systems, while a second insult triggers the full clinical syndrome. The high prevalence of ME/CFS and POTS in post-COVID cohorts reinforces the role of SARS-CoV-2 as a predominant trigger in the current era, suggesting common pathophysiological mechanisms such as autonomic dysfunction, neuroinflammation, and abnormal immune activation [15,16].

The patient's diagnostic journey, which involved multiple specialties and extensive evaluation through complementary tests with normal results, reflects one of the greatest challenges in managing ME/CFS and POTS: the difficulty of applying conventional tests. The absence of specific biochemical or imaging markers often leads to diagnostic delays, disbelief from some healthcare professionals, and additional suffering for the patient. This case highlights the crucial importance of established clinical criteria and functional tests, such as the Tilt Test for POTS and the assessment of PEM for ME/CFS. The diagnosis therefore remains grounded in careful history-taking and recognition of characteristic symptomatic patterns. This reinforces the urgent need for greater clinical training so that healthcare professionals understand that PCCs are real and diverse entities, and must be alert and sensitive to the profile of a significant portion of patients who, like the one described, present with severe illness in the absence of classic laboratory abnormalities.

MCAS was considered as a possible unifying mechanism for the patient's multisystem symptoms, including urticaria, gastrointestinal complaints, intolerances, fatigue, and brain fog. However, no objective testing (serum tryptase, urinary histamine metabolites) was performed or confirmatory in this case. Therefore, MCAS remains an unconfirmed hypothesis and should be interpreted with caution. Growing evidence suggests an association between MCAS and PCC, ME/CFS, and POTS [17,18], but further objective testing is needed before drawing definitive conclusions.

From a therapeutic standpoint, the management of ME/CFS and PCC with POTS is characterized by an empirical and multidisciplinary approach, largely guided by real-world evidence reported by patients. For POTS, this systematic review highlights interventions such as compression stockings and salt supplementation as first-line non-pharmacological approaches that improve symptoms and hemodynamics, while ivabradine and beta-adrenergic blockers (such as propranolol and bisoprolol) showed strong evidence among pharmacological options. In parallel, recent studies on ME/CFS highlight other interventions with consistently positive responses: pacing, considered the strategy with the highest perceived benefit, especially improves fatigue, PEM, and brain fog; low-dose naltrexone leading to significant results in the same symptoms, albeit with a limited effect on POTS; and intravenous or subcutaneous immunoglobulin (IgG) with broad benefits, including for fatigue, post-exertional malaise, POTS, and brain fog, particularly in patients with multisystem symptoms [16,19].

The prescribed medication regimen combined neuromodulators, an anti-inflammatory agent (PEA), and attempts at immune modulation (corticosteroid pulse therapy). It is important to note that the MCAS hypothesis opens a distinct pharmacological perspective. If confirmed, management could include targeted anti-mediator therapy, based on the use of H1 and H2 antihistamines, mast cell stabilizers, leukotriene antagonists, and, in refractory cases, corticosteroids or anti-IgE therapy [20]. This arsenal represents a complementary and specific strategy to modulate mast cell activity, which may underlie many of the patient's symptoms.

A crucial finding, which corroborates the ME/CFS diagnosis, was the clear worsening of symptoms after starting light walks (PEM), a pathophysiological hallmark that contraindicates conventional exercise and demands extremely gradual rehabilitation approaches, such as pacing therapy. Treatment responses were partial and fluctuating, and no objective biomarkers were available to quantify them. This underscores the lack of standardized and effective therapies for these conditions.

Finally, the patient's trajectory highlights the profoundly disabling impact of these syndromes associated with PCC. The repeated work absences, the drastic reduction in daily living and leisure activities, and the persistent impairment in quality of life illustrate that, despite the absence of standardized objective markers in conventional tests, these are conditions with severe functional and socioeconomic consequences. This report reinforces the urgent need for clinical recognition, investment in research for biomarkers and specific treatments, and the implementation of integrated, multidisciplinary care models. Such models are essential to provide adequate support for a growing population of patients who, like the one described here, face the challenges of complex chronic syndromes triggered by viral infections.

## Conclusions

The clinical overlap between PCC, ME/CFS, and POTS, as illustrated in this case, highlights the complex clinical trajectory that may follow SARS-CoV-2 infection. The patient had a history of infectious mononucleosis due to EBV in childhood. The temporal sequence of EBV infection followed years later by SARS-CoV-2 infection is clinically relevant, although this single case cannot determine whether these infections acted as sequential triggers. The temporal association between SARS-CoV-2 infection and the subsequent development of persistent multisystem symptoms supports the clinical characterization of these conditions in the context of post-COVID illness, while not establishing a specific causal mechanism.

The diagnostic difficulty stemming from the absence of conventional biomarkers demands that healthcare professionals and systems develop and adopt standardized clinical protocols, with enhanced clinical training and the adoption of functional criteria, such as the Tilt Test and the assessment of PEM. Given the devastating functional and socioeconomic impact, there is an urgent need for the development of integrated, multidisciplinary care models, alongside investments in translational research to identify biomarkers and targeted therapies.
